# Case report: The gait deviation index may predict neurotherapeutic effects of FES-assisted gait training in children with cerebral palsy

**DOI:** 10.3389/fresc.2023.1002222

**Published:** 2023-03-03

**Authors:** Ahad Behboodi, Aswhini Sansare, Nicole Zahradka, Samuel C. K. Lee

**Affiliations:** ^1^ NAB Laboratory, Rehabilitation Medicine Department, Clinical Center, National Institutes of Health, Bethesda, MD, United States; ^2^ Pediatric Mobility Laboratory, Department of Physical Therapy, University of Delaware, Newark, DE, United States

**Keywords:** functional electrical simulation (FES), CP, neuromuscular electrical stimulation (NMES), neurotherapeutic, motor training, gait training, neuroprosthesis, case report

## Abstract

**Background:**

Children with cerebral palsy (CP) show progressive loss of ambulatory function characterized by kinematic deviations at the hip, knee, and ankle. Functional electrical stimulation (FES) can lead to more typical lower limb kinematics during walking by eliciting appropriately timed muscle contractions. FES-assisted walking interventions have shown mixed to positive results in improving lower limb kinematics through immediate correction of gait during the application of FES, or long-term, persisting effects of non-FES-assisted gait improvements following multi-week FES-assisted gait training, at the absence of stimulation, i.e., neurotherapeutic effects. It is unknown, however, if children with CP will demonstrate a neurotherapeutic response following FES-assisted gait training because of the CP population's heterogeneity in gait deviations and responses to FES. Identifying the neurotherapeutic responders is, therefore, important to optimize the training interventions to those that have higher probability of benefiting from the intervention.

**Objective:**

The purpose of this case study was to investigate the relationship between immediate and neurotherapeutic effects of FES-assisted walking to identify responders to a FES-assisted gait training protocol.

**Methods:**

The primary outcome was Gait Deviation Index (GDI) and secondary outcome was root mean squared error (RMSE) of the lower extremity joint angles in the sagittal plane between participants with CP and a typically developing (TD) dataset. Potential indicators were defined as immediate improvements from baseline during FES-assisted walking followed by neurotherapeutic improvements at the end of training.

**Case description:**

Gait analysis of two adolescent female participants with spastic diplegia (Gross Motor Function Classification System level II and III) was conducted at the start and end of a 12-week FES-assisted treadmill training protocol. Participant 1 had scissoring crouch gait, while participant 2 had jump gait.

**Outcomes:**

The GDI showed both immediate (presence of FES) and neurotherapeutic (absence of FES after training period) improvements from baseline in our two participants. Joint angle RMSE showed mixed trends between immediate and neurotherapeutic changes from baseline. The GDI warrants investigation in a larger sample to determine if it can be used to identify responders to FES-assisted gait training.

## 1. Introduction

Cerebral palsy (CP) is a movement disorder caused by brain injury during fetal or infant development. It is the most prevalent childhood neuromotor diagnosis with an estimate of 764,000 cases in United States (1). While the initial brain injury is non-progressive, musculoskeletal impairments and functional limitations, particularly problems in gait, are progressive (2). About 70%–80% of CP cases are classified as spastic CP (3, 4), characterized by weak and short muscles, muscle spasticity, and impaired selective motor control (5, 6). Children with spastic CP show kinematic deviations at the hip, knee and ankle leading to gait deviations such as crouch gait, jump gait, and equinus gait (7, 8). Walking function in children with CP typically declines from adolescence and into adulthood (9), which contributes to diminished participation in physical activity. Children with disabilities have greater physical activity requirements to prevent decline in their level of function and to prevent secondary conditions that can result from inactivity (10). Consequently, as adults, the complications of chronic sedentary lifestyle significantly increase the level of disablement and decrease quality of life of individuals with physical disability (11).

Due to the high incidence and cost associated with CP, improved rehabilitation strategies for this population are critical (12, 13). Currently, surgical and pharmaceutical treatments for gait disorders are inadequate and often cause further muscle weakness (14). Task-specific gait training interventions, such as body-weight–supported treadmill training, have shown some success in improving gait in individuals with CP (15). Task-specific training may produce longer-lasting effects if combined with interventions that provide physiological-based corrections to reinforce movements such as what occurs with functional electrical stimulation (FES). The goal of FES is to elicit functional movement by applying electrical stimulation to the muscle, and it can be applied in conjunction with volitional movement to facilitate motor learning. By eliciting appropriately timed muscle contractions, FES may compensate for deficits in selective motor control, and thereby, may lead to more typical lower limb kinematics during walking (14). Additionally, by recruitment of Golgi tendon organs and muscle spindles, FES provides rich sensory feedback *via* the afferent corticospinal pathways and conveys proprioceptive and somatosensory information (13). Therefore, FES activates all available sensorimotor components involved in motor control (13) and impacts cortical excitability (14), which may further translate into motor learning. A growing amount of evidence supports that FES improves neuromuscular deficits in spastic CP (14). FES-assisted walking interventions, however, demonstrated mixed results, and thus, there is a strong need for identifying those who may best respond to such interventions, to appropriately prescribe treatment.

FES-induced kinetic and kinematic changes, depending on dosage of intervention and presence of FES, can be divided into three categories: (1) immediate correction in the presence of FES (immediate effect), (2) correction after a period of training in the presence of FES (training effect), and (3) persisting effect without FES, following a multi-session training protocol (neurotherapeutic effects). Note that FES is present when immediate effect is measured, and FES is not present when neurotherapeutic effect is measured. Additionally, neurotherapeutic effects are measured after a period of time following training to demonstrate if FES training can induce lasting corrections, unlike immediate effects which are measured when FES is first applied during walking before practice has taken place (not after first training session). When interpreting the reported results, one should differentiate between training and neurotherapeutic effects as many of the reported outcomes post-training are FES-assisted after a period of training. Whereas we are interested in the non-FES-assisted effects that persist after a period of training with FES.

The positive immediate and training effects of FES-assisted walking interventions in children with CP include improved spatiotemporal gait parameters (16, 17), gait kinematics (17–20), kinetics (20), and foot clearance in the swing phase and at initial contact (17, 19, 21–23). Despite these positive immediate and training effects on gait kinetics and kinematics, evidence of the neurotherapeutic effect has been limited to improvements in clinical measures of mobility, which imply improvements in gait but are not direct measures of gait kinetics and kinematics. For the present study, we focus on the effects that directly translate into kinematic and kinetic improvements as a measure of reduced gait deviations. In a randomized controlled trial in 32 children with CP [mean age = 10 years 8 months, Gross Motor Function Classification System (GMFCS) level I or II], the neurotherapeutic improvements of applying FES to tibialis anterior (TA) muscle were limited to mobility and balance scores and reduced gastrocnemius spasticity (17). In a case series in 14 children/adolescents with CP (mean [SD] age = 13.1 [3.56] years; GMFCS level I or II), the neurotherapeutic effect of applying FES to the TA muscle was increased muscle size (24). The immediate and training improvements were primary effects in both studies; the ankle angle during swing and at initial contact improved in the presence of FES. There were discrepancies between immediate and neurotherapeutic effects in these two studies; the ankle angle improvement with FES was not presented post training without FES. Such omissions demonstrate a challenge in identifying who has a neurotherapeutic response to FES-assisted walking interventions.

Most available FES-assisted walking studies in the CP population are focused on the TA muscle; they strongly suggest immediate and training effects of FES on ankle angle improvement at initial contact and during swing. CP gait deficits, however, are complex and occur throughout the gait cycle; the direction of progress points toward the use of multi-channel FES devices to act across multiple joints (14). For example, stimulation of both rectus femoris and vastus lateralis muscles were more effective in improving posture than stimulation of each muscle alone during walking in one child with CP (25). Three recent multi-channel FES-assisted walking studies demonstrated positive immediate (26, 27) and training effects (28) on joint angles of a total of nine individuals with CP. However, as expected, the improvements are variable across the individuals, joints and phases of gait.

Rose et al. (26), demonstrated positive immediate effects on Gait Deviation Index (GDI) scores, and on joint kinematics during gait when FES was applied. Accordingly, our original hypothesis was that by increasing the multi-channel FES dosage *via* implementing a training intervention, and subject-specific stimulation protocols we may induce neurotherapeutic improvements in gait. We modified their intervention by (1) increasing the dosage of training, (2) devising a subject-specific stimulation protocol, and (3) increasing the number of muscle groups that the system was capable of stimulating. Because the response to FES-assisted walking is influenced by the variation of gait patterns (28) and the subject-specific responses to FES (20, 29), we believe one size FES program does not fit all (26). To address these variabilities, it is necessary to prescribe individualized stimulation programs. Additionally, to add more flexibility in addressing the heterogeneous gait deviations in the CP population, we included most major lower extremity muscle groups, including TA that demonstrated the more consistent positive response to FES. Our group developed a 12-week FES-assisted training program that consisted of a total of 30 min of walking per session, three sessions each week (a total of 36 sessions). A distributed learning model was used consisting of alternating walking with/without FES on a treadmill followed by overground walking three times per week (28). The dosage and the training protocol were based on the FastFES gait training paradigm proposed by Binder–Macleod and colleagues (29). The multi-channel FES system used during training was designed and evaluated by our group (27, 28, 30–32) and included the flexibility to provide individualized stimulation to address subject-specific gait deviations. The FES system had the capability to detect all seven phases of gait and to stimulate up to 5 muscle groups for each leg during each gait phase.

The most desirable outcome of FES training, like any other physical training, is a lasting therapeutic effect, i.e., creating lasting improvements obtained with FES training even in the absence of FES application (neurotherapeutic effect). In an ideal scenario, the effect of FES-assisted gait training creates permanent improvements; individuals would not have to wear the FES system as an orthotic. Considering time commitment associated with training and the inconsistency in neurotherapeutic effects of FES between participants, it is desirable for the therapists to identify who may respond to a high dosage (multi-session) FES intervention and demonstrate immediate improvements. One intuitive indicator might be the immediate response to FES, i.e., the ability to minimize an individual's gait deviations when FES is applied. The purpose of this case study was to quantify the immediate and neurotherapeutic effects of FES-assisted gait training on lower limb kinematics in two individuals with CP, and thereby, investigate the relationship between immediate and neurotherapeutic effects. Here, we hypothesize that participants, who demonstrate immediate kinematic improvements from baseline towards more typical gait (the gait of typically developing children), quantified using metrics such as GDI score, will also have neurotherapeutic improvements towards more typical gait to a high dosage intervention delivered with a multi-channel FES system.

## 2. Methods

### 2.1. FES-assisted walking intervention

A stimulation protocol was developed by three physical therapists (PTs) using videos of each participant's gait to determine the muscles to target (muscle selection: gluteals, hamstrings, quadriceps, dorsiflexors, and/or plantarflexors) and the timing (gait phase) of stimulation to address the participant's gait deviations. Two PTs (PT, PhD) were trained pediatric PTs with expertise in the CP population; the remaining PT had a master's degree in biomechanics and had 8 years of experience as a research PT working with children with CP. The PTs made separate stimulation protocol recommendations based on observations of subject's gait (video) and discussed them. When recommendations differed, a consensus stimulation strategy was defined through discussion by considering the best compromise to improve gait kinematics and walking speed. Stimulation amplitude and pulse duration were set for each muscle group and gait phase, individually, to address subject-specific gait deviations as prescribed by the PTs' evaluation and determined in a procedure called thresholding (27, 28); the thresholding procedure and individualized stimulation protocol for each participant can be found in Appendices A and B, respectively. At any time during training or assessments, if the participant experienced excessive exhaustion or discomfort (verbally communicated) or the operators noticed such behavior, the physical therapist stopped the treadmill immediately with an emergency stop button. The participants were trained using their specific protocols for 12 weeks 3 times per week and 1 h per session. Post-training both participants demonstrated neurotherapeutic functional and kinematic improvements, which included improved walking distance as measured by 6 min Walk Test. Detailed results of this intervention are published in our previous work (28).

### 2.2. Participants

Two female participants with spastic diplegia took part in our FES training program. Participant 1 (CP01:12 years and six months old, GMFCS level III) had a scissoring crouch gait and used a rollator for ambulation. Scissoring crouch gait is characterized by excessive hip and knee flexion with excessive adduction and/or internal rotation at the hip, leading to the lower limbs crossing over in a scissor-like pattern (33). Her initial gait analysis revealed increased hip flexion throughout gait, increased knee flexion from mid-swing through mid-stance, and increased ankle dorsiflexion throughout most of the gait cycle. Participant 2 (CP02: 13 years five months old, GMFCS level II) had a jump gait and did not use an assistive device. Jump gait is characterized by plantarflexion at the ankle, hip and knee flexion, and anterior tilt and excessive lumbar lordosis at the pelvis (34). Her baseline gait analysis showed increased hip flexion throughout the gait cycle, increased knee flexion from mid-swing through terminal stance, and nearly normal ankle dorsiflexion.

The study was approved by the University of Delaware and Shriners Hospitals for Children, Philadelphia (Western Institutional Review Board). Assent and consent were obtained from the participants and their parents, respectively, prior to the study.

The worksheet provided by Schwartz et al. (35) was used to generate a comparator group. The 166 typically developing subjects (TDs) included in this sheet were used as our control group. In this study, the direction of kinematic improvements was defined as getting closer to those measured for the TD group.

### 2.3. Motion capture data collection (assessment)

Kinematic (sampling rate 128 Hz) and kinetic [two force places (Bertec, Columbus, OH, United States) with a sampling rate of 3,200 Hz] data were collected, for the assessment, using instrumented motion capture (Motion Analysis Corp., Santa Rosa, CA, United States) while patients walked at their initial self-selected walking speed on an instrumented treadmill (Bertec Corp, Columbus, OH, United States). Kinematic data were averaged over all complete gait cycles (from 15 to 30 cycles) and were analyzed in Visual 3d (C-Motion Inc., Rockville, MD, United States). Data were normalized to a gait cycle (%) defined by consecutive heel strikes on the treadmill's force plates.

**Figure 1:**
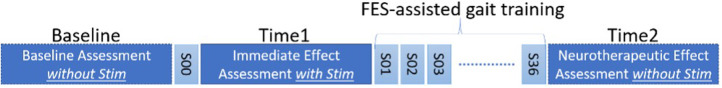
Assessment timeline. S00 is a single FES-assisted walking session before the FES-assisted gait training for 12 weeks (36 sessions, S01 to S36).

Three time points were assessed in children with CP: (1) Participants walking kinematics, at the baseline session, S00 in Figure 1, before application of FES assistance; (2) at the same session during the application of FES before the start of the 12-week FES-assisted gait training (S01-S36 in Figure 1), at Time1 (immediate effect), and (3) after the conclusion of the FES-assisted gait training without the presence of FES at Time2 (neurotherapeutic effect) (Figure 1).

### 2.4. Gait deviation index (GDI)

The GDI was the primary outcome measure and compared with the control dataset at baseline, Time1 and Time2 to examine if FES led to a shift towards the gait of typically developing children in each participant.

GDI is a comprehensive index of gait pathology based on 15 different kinematic measures (i.e., hip motion in three planes, pelvic motion in three planes, foot progression, and ankle and knee angles in the sagittal plane), to quantify the similarity of our participants' gait to the that of control individuals measured by the scaled distance of the kinematics measures from those of control subjects (35). An increase of 5.0 points is considered a clinically relevant improvement in GDI (26); the GDI of normal gait is 100 ± 10 (26). Every 10-point decrease from a GDI score of 100 is one standard deviation from the control individuals (35).

### 2.5. Root mean square error (RMSE)

**Figure 2:**
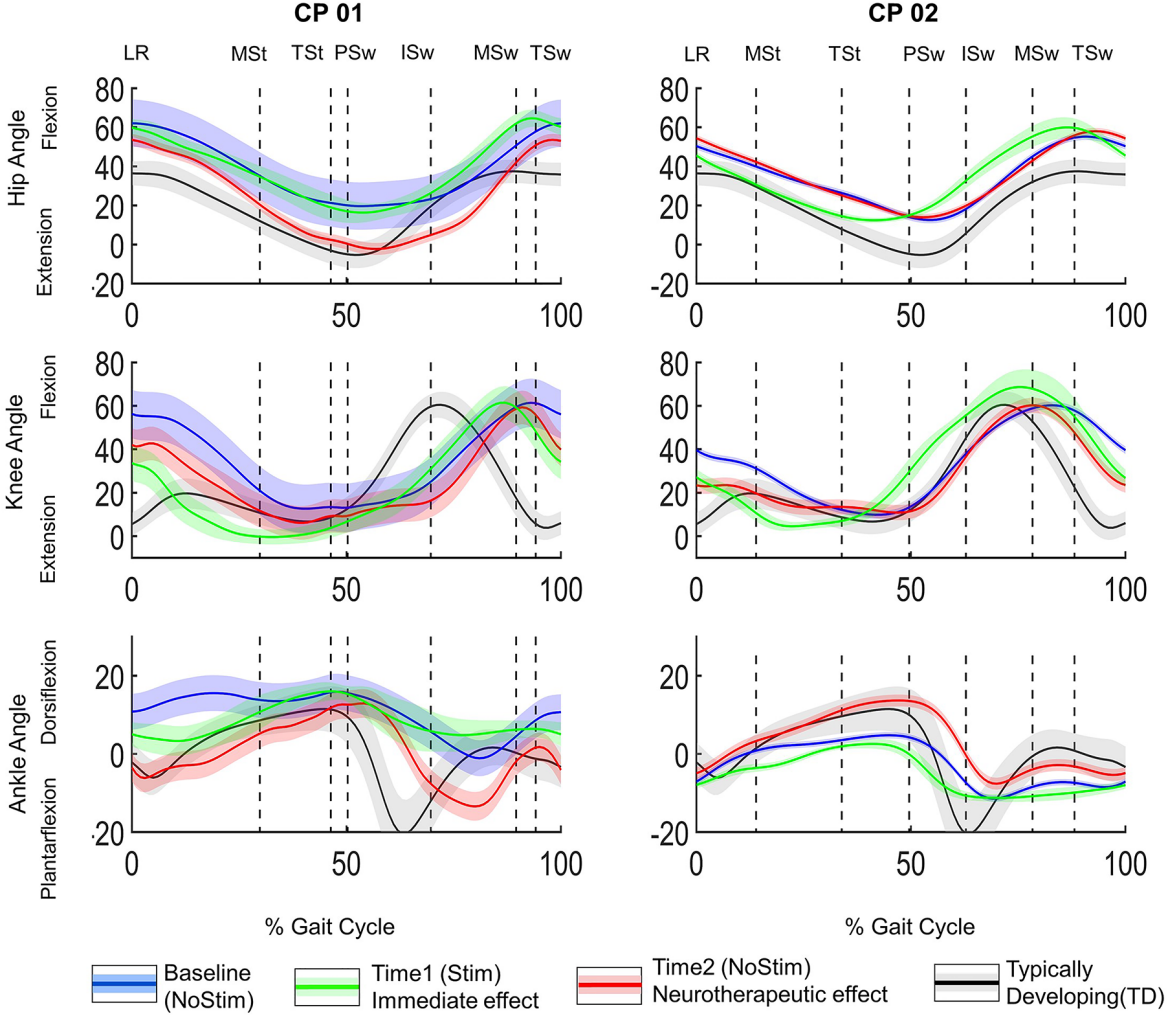
Joint angles normalized to gait cycle. Hip, knee and ankle angles for CP01 (GMFCS level III) and CP02 (GMFCS Level II) in the left and right panels, respectively. The three conditions evaluated were (1) before application of FES = baseline (NoStim) in blue, (2) Time1, during FES-assisted walking = immediate effect in green, and (3) after 12-week FES-assisted training protocol, without stimulation (NoStim)= neurotherapeutic effect (Time2). The black traces are the joint angles from GDI worksheet normative dataset of typically developing subjects (TD). Gait phase is indicated by the vertical lines. LR, loading response, MSt, midstance, TSt, terminal stance, PSw, pre-swing, ISw, initial swing, MSw, midswing, TSw, terminal swing.

The secondary outcome measure was root mean square error (RMSE) between the participant's hip, knee, and ankle joint angles (in the sagittal plane, Figure 2) and the average joint angles of the aforementioned TDs in the sagittal plane were used for RMSE calculation (black trace, Figure 2). An increase in RMSE implies a greater deviation from typical gait.

GDI and RMSE were calculated for both sides in Tables 1, 2, respectively. For simplicity, only the data from the more affected side was included in the visualization of the sagittal plane joint angles (Figure 2). Note that the left side was the more affected side for both participants.

**Table 1:** Left and right GDI score before applying FES at baseline (without stimulation), at Time1 (with stimulation) during FES-assisted walking before the training intervention (Time1), and at Time2 after 12-week FES-assisted gait training, at the absence of stimulation (non-FES-assisted walking).

	Baseline (NoStim)		Immediate effect Time1 (Stim)		Neurotherapeutic effect Time2 (NoStim)	
	Left	Right	Left	Right	Left	Right
CP01	52.14	54.96	56.04	56.59	63.53	63.66
CP02	65.84	71.46	67.20	74.97	68.01	71.88

**Table 2:** Root mean square error (RMSE) of joint angles between TD dataset and participants with CP at baseline: no simulation, time 1: immediate application of FES during walking, and time 2: conclusion of training with FES off (non-FES-assisted walking).

		RMSE				Changes from Baseline			
Joint	TD compared to	CP01		CP02		CP01		CP02	
		Left	Right	Left	Right	Left	Right	Left	Right
Hip	Baseline	18.4	20.6	11.8	12.9	–	–	–	–
	Time1	19.9	22.9	17.1	15.1	1.5	2.3	5.3	2.2
	Time2	11.5	11.1	16.0	14.2	−6.9	−9.5	4.2	1.3
Knee	Baseline	26.4	27.0	18.3	10.5	–	–	–	–
	Time1	20.7	19.5	16.7	15.0	−5.7	−7.5	−1.6	4.5
	Time2	24.9	22.6	11.6	4.2	−2.5	−4.4	−6.7	−6.3
Ankle	Baseline	13.0	13.8	8.1	7.7	–	–	–	–
	Time1	9.3	8.7	6.3	6.1	−3.7	−5.1	−1.8	−1.6
	Time2	10.5	10.3	6.9	8.1	−2.5	−3.5	-1.2	0.4

Changes from baseline is calculated by subtracting Time1 and Time2 RMSEs from baseline (Time – Baseline); thus, the negative RMSE changes was considered improvement. The highlighted cells in red (under the “Changes from Baseline”) demonstrated opposite trends of change from the baseline.

## 3. Results

### 3.1. GDI

The **left side GDI** scores of both subjects showed positive trends toward TD from baseline at Time1 and Time2. CP01 (GMFCS level III) demonstrated clinically relevant improvement at Time2, an increase of about 11 points from baseline. The improvement in CP02 (GMFCS level II) was smaller when compared with CP01, a total of 2.17 points. The **right side** GDI for CP01 demonstrated similar trends and levels of improvements to the left side; for CP02, although the trends from baseline were positive at both Time1 and Time2, the neurotherapeutic improvement was only 0.42 point at Time2.

### 3.2. RMSE

**CP01:** Neurotherapeutic improvements were observed in the RMSE of all three joints, i.e., RMSE decreased between baseline and Time2. This decreasing trend, however, was not reflected in the immediate changes of the hip angle between baseline and Time1; the hip RMSE increased 1.5° (from 18.4° to 19.9°) and 2.3° (from 20.6° to 22.9°) from the baseline at Time1 for the left and right sides, respectively. **CP02:** The immediate effect (baseline to Time1) and the neurotherapeutic effects (baseline to Time2) demonstrated similar decreasing or increasing trends in all of the joints except right knee and ankle. The increase of 4.5° in the right knee RMSE at Time1 was in the opposite direction of neurotherapeutic effect, a decrease of 6.3° from baseline in the knee RMS at Time2; for right ankle RMSE, a decrease of 1.6° from baseline at Time1 was in contrast to 0.4° of increase from baseline at Time2. All these RMSE changes and the joint angles at each time points are demonstrated in Table 2. The cells that are highlighted in red are the time points that are demonstrating opposing trends of change from baseline.

### 3.3. The visual inspection of saggital plane joint angles

In **CP01**, the hip was almost unchanged compared to the baseline at Time 1 (green trace), however, it got more extended, typical, at Time2 (red trace in Figure 2). During the initial swing, the ankle was more dorsiflexed than the TD (black trace) at Time1, however, it became more plantarflexed than TD after the training (red trace). In **CP02**, the hip was more extended, during the stance, at Time1, i.e., more typical; it returned to the baseline (blue trace) at Time2. The knee was more flexed during pre-swing, i.e., less typical, at Time1; it returned to its baseline pattern, a more typical pattern, after the training (red trace). Ankle: except for the loading response, the ankle was less typical throughout the gait cycle at Time1; however, its pattern followed the TD pattern (black trace) for most of the gait cycle at Time2 and was substantially more dorsiflexed during the gait cycle.

## 4. Discussion

We explored the potential to use acute kinematic changes measured by RMSE and GDI score, during the first session of applying FES, as indicators of the neurotherapeutic effects following training with FES in two participants with CP. The hypotheses were that the direction of change in the GDI and RMSE were the same for the immediate effect (change between baseline and Time1) and neurotherapeutic effect (change between baseline and Time2). Thereby, one could use the immediate changes (baseline to Time1) to identify individuals who would have neurotherapeutic changes (baseline to Time2). The improvement in GDI scores of both sides at Time1, the immediate effect of FES (Stim), could indicate improvement at Time2 when compared to the baseline, i.e., the neurotherapeutic effect of FES (NoStim). The trend of changes in the joint angles RMSE, on the other hand, was inconsistent. Therefore, GDI was a potential indicator of the neurotherapeutic effect of FES, using its immediate effect. Note that, our hypothesis was regardless of the magnitude of immediate and neurotherapeutic changes; ideally the magnitude of neurotherapeutic improvements would be larger than immediate improvements meaning the trend of improvement would be ascending from baseline to Time1 and continues with further improvements from Time1 to Time2, i.e., basline < Time1 < Time2.

GDI has previously been reported to be a reliable measure and was used in many studies (23, 26, 36–38) for assessing the similarities of the pathological gait to a desired trajectory, e.g., a dataset of 166 TDs (35). By including 15 kinematic measures from all three joints, GDI is an inclusive kinematic measure and can capture this interdependency, and thereby, the lower extremity kinematics changes, more thoroughly when compared with joint-by-joint analysis. For more affected side, the GDI improvements from baseline to Time1 (immediate effect) not only could predict the improvement between baseline and Time2 (neurotherapeutic effect) in both participants, but also the trend of improvements between baseline and Time1 were followed by further improvements between Time1 and Time2. For the CP02's less affected side, however, the GDI improvement between baseline and Time1 was not followed by further improvements at Time2. The improvement between baseline and Time1 (immediate effect), however, could predict the improvement between baseline and Time2 (neurotherapeutic effect). Therefore, if tested thoroughly using more subjects and similar consistency in the trend of kinematic changes, GDI might be a reliable measure to identify those who would show improved outcomes after FES-assisted gait training, especially for the more affected side.

It is worth noting that the magnitude of the GDI changes might be different from subject to subject, potentially depending on the level of mobility. As can be seen, CP01 (GMFCS III) had more substantial and clinically relevant changes toward TD, more than one standard deviation (11 points). This might be because of lower CP01's GDI score at the baseline (52.14), and thus more capacity for improvement, when compared with that of CP02 (65.84), i.e., CP01 was almost 5 SD away from TD in comparison to 3.5 SD for CP02. Similar discrepancies in the magnitude of improvements were reported previously for these two subjects (28). These results suggest the need for further FES-assisted walking interventions on CP population with GMFCS level III. The FES-walking studies on CP, however, are mostly focused on GMFCS levels I and II (14).

When joint-by-joint RMSE was used as the indicator, the immediate effects were not necessarily in the same direction as neurotherapeutic effects; therefore, our hypothesis was rejected for this measure. For example, although the knee and ankle RMSEs improved (decreased) at Time2 for more affected side, in both subjects, but they demonstrated detrimental immediate effect at Time1, i.e., RMSE change away from TD and thereby, RMSE increase between baseline and Time1 for CP02's right ankle and knee (Table 2). Thus, one cannot identify the direction of neurotherapeutic changes using joint by joint RMSE changes from baseline at Time1 (immediate effect). GDI, on the contrary, captures overall lower extremity kinematic changes, and might be an alternative measure for such predictions.

The visual inspection of the joint angles in the sagittal plane (Figure 2) further demonstrated the heterogeneous kinematic changes. Because of the complexity of these changes, one may not be able to use visual inspection of joint angles to predict the neurotherapeutic effect of FES-assisted walking interventions. For example, at Time2 (red trace in Figure 2) the CP02's ankle angle, improved from the Loading Response to the beginning of Pre-swing; then, moved away from TD pattern (black trace) through the beginning of the Midswing and then get closer to the TD pattern again through the end of the gait cycle. Different joints also responded differently to FES, within a subject. For example, in CP01 at Time2 hip and ankle improved toward TD patterns, when compared to the baseline (blue trace in Figure 2), in all of the gait phases except Initial Swing; the knee changes, however, were mixed during the gait cycle. Thus, when assessing the kinematic improvements of such interventions, there is a need for a more comprehensive method such as GDI and RMSE that takes into account the similarity of the overall pattern of joint angle while including all lower limb joints and different axes.

The main limitation of this study was the number of participants in our training protocol. More subjects are needed to find a reliable indicator for identifying the responders for our FES-assisted gait training protocol. Additional comprehensive gait metrics, such as Movement Deviation Profile (39) and Gait Variable Score (GVS), which is derived from Gait Profile Score (GPS) (40), must be assessed to identify a robust and sensitive metric for predicting the trend of kinematic changes or even potentially its magnitude. Note that GPS and GDI were shown to be highly correlated; therefore, GVS might demonstrate similar trends to GDI (41). Additionally, because kinematics of overground walking could be different that those of treadmill walking, and improvements in overground walking is the overarching goal of treadmill gait training, the predictability of candidate metrics must be tested overground.

## 5. Conclusion

As the main objective of this study, we investigated if the acute kinematic changes of FES application during walking can be used as a potential indicator of changes following long-term FES-assisted gait training, and thereby, to identify the responders. To this end, two kinematic-based measures were evaluated. The GDI score changed in the same direction between baseline vs. Time1 and baseline vs. Time2 for both participants; therefore, the direction of changes at Time1 may be an indication of the direction of changes after a period of FES-assisted gait training. The immediate changes from baseline in RMSE did not follow the same trend post-training for all joint angles. Additionally, predicting the neurotherapeutic effects by visual inspection of the immediate changes in joint angles can be complicated, as expected, without a clear indication if FES-assisted gait training is helpful. Regardless, with continued training, FES assistance appears to be beneficial in reducing gait deviations, as indicated by improvements made in the GDI. Therefore, an inclusive measure, GDI, which includes the kinematics from all three lower extremity joints, may be a candidate measure for indicating the neurotherapeutic effect of FES using its immediate effect; more participants, however, are required for any conclusion.

## Data Availability

The raw data supporting the conclusions of this article will be made available by the authors, without undue reservation.
