# Supplementary material for: Case report: The gait deviation index may predict neurotherapeutic effects of FES-assisted gait training in children with cerebral palsy
Source: Front Rehabil Sci. 2023 Mar 3;4:1002222. doi: 10.3389/fresc.2023.1002222 (PMC10020343; doi:10.3389/fresc.2023.1002222)
Supplement: Supplementary file 1 [file Datasheet1.pdf]

APPENDIX A: FES THRESHOLDING [1]

FES Thresholding is the process for determining the values of stimulation intensity, i.e., electrical stimulation pulse width and current, necessary to achieve sufficient muscle contractions to produce phase-specific functional goals (e.g., to achieve dorsiflexion during swing). To this end, we broke down the functional goal of walking for each muscle group in each gait phase (**Figure A1**).

Establishing a baseline intensity to produce a motor response. For each muscle, while the subject was standing in the neutral position, the stimulation current was initially set to 30 mA and pulse duration was gradually increased from zero in increments of 5 µs until a strong twitch, i.e., motor response, was observed. If pulse duration reached 250 µs before the motor response was observed, the current was increased by 10 mA and pulse duration was again incrementally increased from 100 µs. The pulse frequency was constant at 40 Hz for the whole protocol.

Fine-tuning the intensity to produce the functional response. For each gait phase, with the subject's legs positioned to mimic the functional goal in the phase, stimulation parameters were set at baseline threshold values of the phase, and each muscle group was stimulated in turn using a sequence of pulses at 40 Hz. The time duration of outputting the train of pulse was set to a length of time corresponding to the subject-specific gait phase duration (typically 50-300 ms). Pulse duration was incrementally increased by 5 µs until the functional goals were met or the subject's maximum tolerance was reached. If the maximum pulse duration (500 µs) was reached before achieving the desired functional goals, the stimulation current was increased by 10 mA and pulse duration was incrementally increased, again, from the baseline value. Clinical judgment was used to determine if the functional goals were met (**Figure A1**).

Threshold values were reevaluated visually by a physical therapist during treadmill walking to ensure that the desired functional responses were achieved while the subject was in motion. Clinical judgment and subject feedback were used to make the final adjustments to the stimulation intensity by modulating the pulse duration to determine final stimulation parameter values. Simulation parameter values were re-validated during treadmill walking at each session and adjusted if necessary. The most recent parameters were used at the beginning of each successive training session.

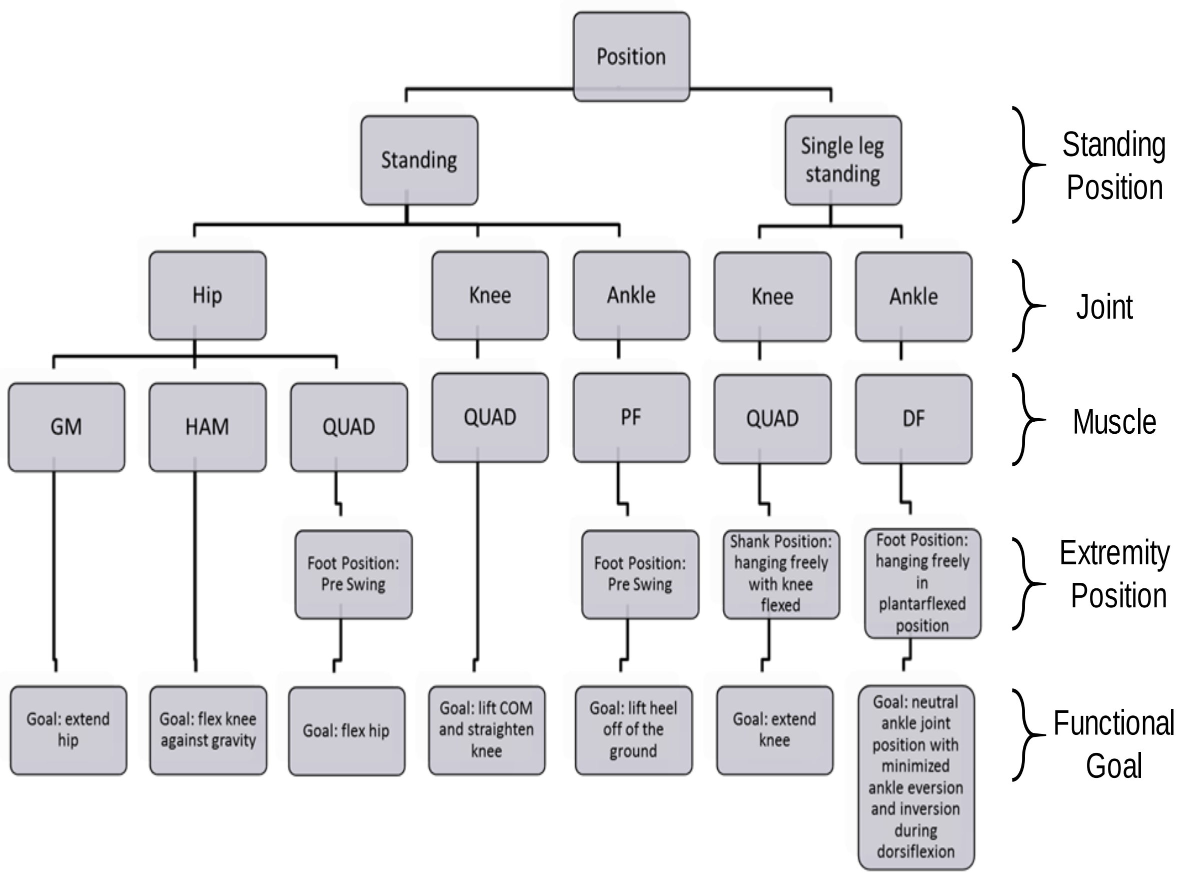

Figure A1. The stimulation thresholding flowchart. For determining the stimulation intensity (pulse width and current), the chart indicates the subject's standing position, the target joint and the muscle group, a secondary position (if necessary), and the functional goal the muscle group should accomplish. Muscles targeted for stimulation include the gluteus maximus (GM), hamstrings (Ham), quadriceps femoris (Quad), ankle plantarflexors (PF) and ankle dorsiflexors (DF).

[1] A. Behboodi, N. Zahradka, J. Alesi, H. Wright, and S. C. K. Lee, "Use of a Novel Functional Electrical Stimulation Gait Training System in 2 Adolescents with Cerebral Palsy: A Case Series Exploring Neurotherapeutic Changes," *Phys. Ther.*, vol. 99, no. 6, pp. 739–747, Jun. 2019.
