# Supplementary material for: Case report: The gait deviation index may predict neurotherapeutic effects of FES-assisted gait training in children with cerebral palsy
Source: Front Rehabil Sci. 2023 Mar 3;4:1002222. doi: 10.3389/fresc.2023.1002222 (PMC10020343; doi:10.3389/fresc.2023.1002222)
Supplement: Supplementary file 2 [file Datasheet2.pdf]

APPENDIX B: INDIVIDUALIZED STIMULATION PROTOCOL [1]

For each subject, the objectives that the prescribed stimulation protocol was supposed to accomplish are explained below:

CP 01 stimulation protocol was prescribed for increasing push-off power, toe clearance in the swing period, stability during weight bearing, and hip and knee extension in the stance period. Because the subject could not tolerate the stimulation on the gluteals muscles, we removed this muscle from the stimulation protocol (Figure B1-a).

CP 02 stimulation protocol was prescribed for increasing push-off power, toe clearance in the swing period, hip extension in the stance period, and knee extension in the swing period, and to pre-position the leg better for weight-bearing portion of the Loading Response (Figure B1-b).

Note that for each muscle, the current (mA) was fixed throughout the gait cycle; its intensity is depicted by blue color. The deeper the blue, the more intense the stimulation current. The pulse width of each muscle, however, was specific to each phase of gait, depicted by red. Black indicates zero pulses or zero current intensity.

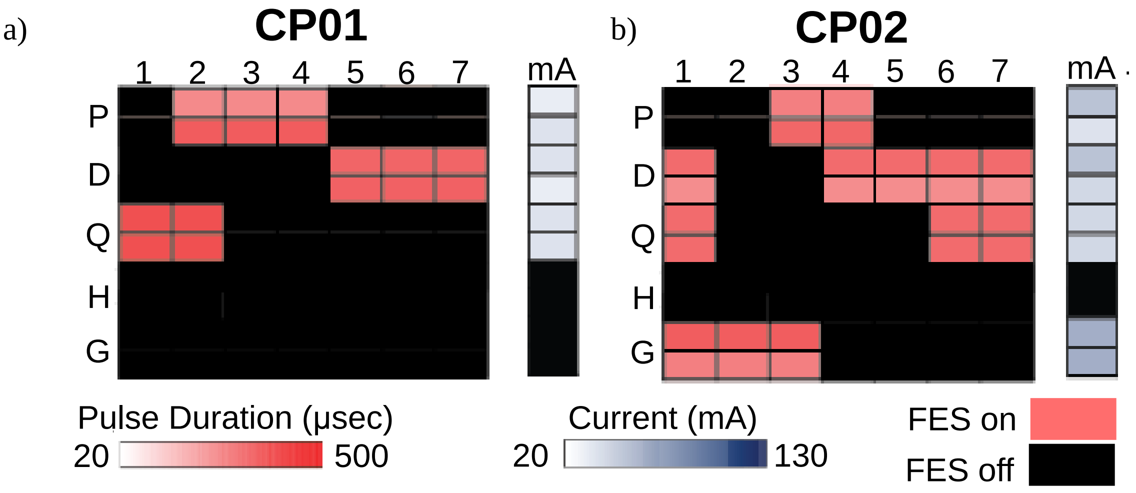

Figure 1. Stimulation protocol for subjects CP01 and CP02. Which muscles (P = Plantarflexors; D = Dorsiflexors; Q = Quadriceps; H = Hamstrings; G = Gluteus Maximus) needs to be stimulated during each gait phase (1= Loading Response; 2= Mid Stance; 3= Terminal Stance; 4= Preswing; 5= Initial Swing; 6 = Mid Swing; 7= Terminal Swing).

[1] N. Zahradka, A. Behboodi, A. Sansare, and S. C. K. Lee, "Evaluation of Individualized Functional Electrical Stimulation-Induced Acute Changes during Walking: A Case Series in Children with Cerebral Palsy," 2021, doi: 10.3390/s21134452.
